# Investigation of the “Not Better Explained” Diagnosis Criteria in Sleep Disorder Classifications: A Systematic Content Analysis and Critical Review

**DOI:** 10.1111/jsr.70327

**Published:** 2026-03-13

**Authors:** Jean‐Arthur Micoulaud‐Franchi, Vincent P. Martin, Julien Coelho, Régis Lopez, Pierre‐Alexis Geoffroy, Laure Peter‐Derex, Isabelle Lambert, Charles M. Morin, Clélia Quiles, Christophe Gauld

**Affiliations:** ^1^ University Sleep Clinic, Services of Functional Exploration of the Nervous System, University Hospital of Bordeaux, Place Amélie Raba‐Leon Bordeaux France; ^2^ Université de Lorraine, CNRS, Inria, LORIA Nancy France; ^3^ National Reference Centre for Orphan Diseases, Narcolepsy‐Rare Hypersomnias, Sleep Unit, Department of Neurology, CHU Montpellier, Univ Montpellier Montpellier France; ^4^ Inserm, U1061, Université Montpellier Montpellier France; ^5^ Département de Psychiatrie et D'addictologie AP‐HP, GHU Paris Nord, DMU Neurosciences, Hopital Bichat—Claude Bernard Paris France; ^6^ Centre Chronos, GHU Paris—Psychiatry & Neurosciences Paris France; ^7^ Université de Paris, NeuroDiderot, Inserm Paris France; ^8^ CNRS UPR 3212, Institute for Cellular and Integrative Neurosciences Strasbourg France; ^9^ Center for Sleep Medicine Hospices Civils de Lyon (HCL), Lyon 1 University Lyon France; ^10^ Université Claude Bernard Lyon 1 CNRS, Inserm, Centre de Recherche en Neurosciences de Lyon CRNL U1028, UMR5292, PAM Team Bron France; ^11^ APHM, Timone Hospital, Sleep Unit, Epileptology and Cerebral Rhythmology Marseille France; ^12^ INSERM, INS, Inst Neurosci Syst, Aix Marseille Univ Marseille France; ^13^ Centre D'étude des Troubles du Sommeil Centre de Recherche CERVO Quebec Canada; ^14^ École de Psychologie, Université Laval Québec Canada; ^15^ Department of University Psychiatry Centre Hospitalier Charles Perrens Bordeaux France; ^16^ SANPSY, CNRS UMR 6033, Place Amélie Raba‐Leon Bordeaux France; ^17^ Department of Child and Adolescent Psychopathology CHU de Lyon Lyon France; ^18^ Institut des Sciences Cognitives Marc Jeannerod, UMR 5229 CNRS & Université Claude Bernard Lyon Lyon France

**Keywords:** diagnostic criteria, DSM‐5, exclusion criterion, ICSD‐3, sleep disorders

## Abstract

Accurate diagnostic boundaries between normal and pathological sleep are essential for appropriate care. Among diagnostic criteria, the “Not Better Explained” (NBE) exclusion criterion prevents misclassification by ensuring that symptoms are not attributable to another disorder. Despite its importance, the NBE criterion has never been systematically analysed across the International Classification of Sleep Disorders—Third Edition Text Revision (ICSD‐3‐TR) and the Diagnostic and Statistical Manual of Mental Disorders—Fifth Edition Text Revision (DSM‐5‐TR). A systematic content analysis was conducted using a validated methodology for diagnostic criteria evaluation. Ten major sleep disorders were selected based on prevalence and clinical relevance. For each disorder in both classifications, NBE formulations were identified, extracted, and categorised by excluded condition and causal wording. The Jaccard index quantified overlap, and visualisations compared exclusion patterns. The ICSD‐3‐TR included the NBE criterion in 9 of 10 disorders, whereas the DSM‐5‐TR included it in 7 of 10. Overall overlap was strong (Jaccard index = 0.75). Differences were mainly semantic. The ICSD‐3‐TR more often excluded “sleep disorders,” “substance use,” and “sleep behaviours,” while the DSM‐5‐TR emphasised “medical” and “mental disorders.” The ICSD‐3‐TR predominantly used “not better explained,” whereas the DSM‐5‐TR employed more variable formulations (“not attributable,” “not adequately explained”…). By highlighting the strengths and inconsistencies in the use of the NBE criterion, we aim to contribute to a more empirically and conceptually grounded framework for sleep disorder diagnosis. Our work aims to inform ongoing efforts to improve the reliability, validity, and clinical utility of diagnostic classifications in sleep medicine.

## Graphical abstract

The “Not Better Explained” (NBE) criterion plays a key role in preventing diagnostic misclassification by structuring causal reasoning in sleep medicine. This systematic content analysis compares the use, wording, and excluded conditions of NBE criteria across the ICSD‐3‐TR and DSM‐5‐TR for ten major sleep disorders. While overall overlap is strong, important differences emerge in exclusion logic and semantic formulation. These differences have direct implications for diagnostic precision, management of comorbidity, and the development of mechanism‐based therapeutic strategies.

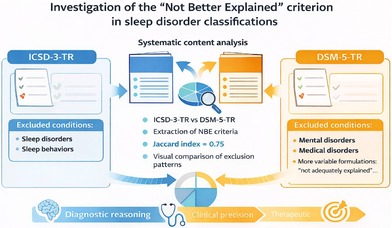

## 1 Introduction

Sleep disorders are common and can lead to significant impairments in health, quality of life, and daytime functioning (The 2022). Epidemiological studies consistently report a high prevalence of sleep complaints across populations, including insomnia and hypersomnolence symptoms (Perez‐Carbonell et al. 2022; Perlis et al. 2022). However, the clinical approach to sleep disorders remains challenged by both the risk of overmedicalisation (where normal variations in sleep are pathologised) and undermedicalisation (where sleep disorders remain unrecognised or misclassified) (Gauld, Lopez, Philip, et al. 2022). Clear and clinically accurate boundaries between normal and pathological sleep are therefore essential to ensure appropriate care and delivery of interventions that are truly meaningful. As with other areas of medicine, establishing reliable and valid diagnostic criteria requires not only empirical rigour but also conceptual precision (Wakefield 1992). In the field of sleep medicine, this task is particularly complex and necessitates a high level of expertise (Martin et al. 2024), given the heterogeneity of symptoms, the multidisciplinary nature of this medical specialty, the variety of causes underlying sleep disturbances, and the frequent overlap with both mental and physical disorders (Pevernagie 2021).

To address this complexity, our research group has conducted a series of systematic investigations into the diagnostic architecture of sleep disorder classifications. We began by analysing the diagnostic criteria outlined in the third edition of the *International Classification of Sleep Disorders* (ICSD‐3), identifying the types of criteria used and the principles underlying their organisation (Gauld, Lopez, Geoffroy, et al. 2021). In this first study, we developed a method for labeling and categorising all 241 criteria across the 43 main sleep disorders in the ICSD‐3. These criteria were classified into nine distinct types: clinical manifestations (i.e., symptoms), objective markers (i.e., a criteria objectifying a sleep related physiological modification), distress and disability (i.e., clinical significance criteria), excluded conditions, duration, frequency, age of onset, and associated conditions (Gauld, Lopez, Geoffroy, et al. 2021). This labeling allowed us to assess the underlying epistemological assumptions guiding sleep nosology, particularly the *harmful dysfunction analysis* (HDA) framework proposed by Wakefield (Wakefield 1992; Gauld, Wakefield, and Micoulaud‐Franchi 2025).

Among the clinical diagnosis criteria used to define a sleep disorder in a reliable and valid way, three overarching categories are particularly important (Gauld, Lopez, Philip, et al. 2022; Gauld, Lopez, Geoffroy, et al. 2021): symptomatic criteria, clinical significance criteria (CSC), and exclusion criteria, the latter often referred to under the label “not better explained by another condition”. Concerning symptomatic criteria, we have conducted analyses of these criteria in the ICSD‐3 and in the *Diagnostic and Statistical Manual* (DSM‐5) sleep disorder sections, using methods such as content analysis and symptom network modelling (Gauld, Lopez, Morin, et al. 2022, 2021). These studies revealed a symptomatic structure in which insomnia and hypersomnolence symptoms form central hubs in the symptom networks, suggesting core dimensions of sleep disorders that transcend traditional diagnostic boundaries (Association of Sleep Disorders Centers 1979). Concerning CSC, we have compared the presence and formulation of the CSC across the ICSD‐3‐TR and DSM‐5‐TR, demonstrating variable inclusion and limited overlap between classifications (Jaccard index = 0.40) (Gauld, Martin, et al. 2025). This variability raises important questions about the role of the CSC in refining the reliability and validity of sleep disorder diagnoses, particularly when applied inconsistently across classifications. Indeed, CSC aims to distinguish pathological conditions from normal variants by requiring that symptoms produce significant distress or disabilities (harms) (Gauld, Martin, et al. 2025).

These previous works on the systematic investigations into the diagnostic architecture of sleep disorder classifications revealed one important criterion that has remained largely unexplored in sleep medicine: the exclusion criterion, aka the “not better explained” criterion (NBE). This diagnosis criterion has an essential exclusionary role in both the ICSD and DSM frameworks, ensuring that a diagnosis of a sleep disorder is only made when symptoms cannot be better attributed to another sleep, mental, medical, or substance‐induced conditions (Gauld, Lopez, Philip, et al. 2022; Gauld, Wakefield, and Micoulaud‐Franchi 2025). In other words, it acts as a safeguard against misclassification by demanding diagnostic parsimony (Slade and Andrews 2002). However, to our knowledge, no comprehensive systematic content analysis of this diagnosis criterion for sleep disorders, across ICSD‐3‐TR and DSM‐5‐TR, has been conducted. This omission is surprising given the central role that NBE criteria play in psychiatric diagnostic systems (Boyd et al. 1984). Indeed, the philosophical and empirical significance of such exclusion criterion has been highlighted in psychiatry as a means of improving construct validity, inter‐diagnostic boundaries, and reliability of diagnoses (Kendler 2018; Zimmerman et al. 2006). Its presence or absence may dramatically affect prevalence estimates, comorbidity rates, and, by extension, the allocation of appropriate therapeutic resources.

In this context, the primary aim of this new work is to conduct a systematic evaluation of the use and structure of the NBE criterion in the current major classifications of sleep disorders: the ICSD‐3‐TR (American Academy of Sleep Medicin 2023) and the DSM‐5‐TR (American Psychiatric Association 2022). Our objective has two main components: (i) to identify and extract all formulations of NBE criterion across the two classifications; (ii) to compare its frequency and wording across different sleep disorder categories, notably by producing a visualisation of these patterns using quantitative and graphical methods. Based on this comparison, we propose avenues for greater coherence in future iterations of sleep disorder classifications. By highlighting the strengths, inconsistencies, and potential omissions in the use of this criterion, we aim to contribute to a more empirically and conceptually grounded framework for sleep disorder diagnosis. Our work aims to inform ongoing efforts to improve the reliability, validity, and clinical utility of diagnostic classifications in sleep medicine.

## 2 Methods

### 2.1 Objective and General Design

A systematic content analysis, based on our previously established methodology for examining diagnostic criteria of sleep disorders (Gauld, Martin, et al. 2025), was conducted to identify, extract, and compare the formulations of the “Not Better Explained” (NBE) criterion across the ICSD‐3‐TR (American Academy of Sleep Medicin 2023) and DSM‐5‐TR (American Psychiatric Association 2022). The analysis aimed to quantify and visualise its distribution and usage in both classifications.

### 2.2 Selection and Labeling of Main Sleep Disorders

Ten major sleep disorders were selected based on their centrality in the evolution of sleep medicine (Schulz 2022), their high prevalence (Ohayon 2011), and their importance as public health and clinical care issues (Hossain and Shapiro 2002), particularly in light of the challenges of both overmedicalisation and undermedicalisation (Gauld, Lopez, Philip, et al. 2022): Chronic insomnia disorder, Narcolepsy, Idiopathic hypersomnia, Obstructive Sleep Apnea and Hypopnea (OSAH), Central Sleep Apnea and Hypopnea (CSAH), Circadian rhythm sleep–wake disorder (CRSWD), Disorders of arousal (DA), Rapid Eye Movement Sleep Behaviour Disorder (RBD), Nightmare disorder, and Restless Legs Syndrome (RLS). Labeling terminology was aligned with the ICSD‐3‐TR (American Academy of Sleep Medicin 2023), as the terminology is not necessarily consistent between the ICSD‐3‐TR and the DSM‐5‐TR. To address this, we relied on the transcoding matrix we previously developed to harmonise the wording of sleep disorders across the selected classifications in our earlier publication (Gauld, Martin, et al. 2025).

### 2.3 Identification and Extraction of the NBE Criterion

For each of the ten sleep disorders in the two classifications, we systematically reviewed the diagnostic criteria to identify the presence of NBE criteria. We defined the NBE criterion as any clause within the diagnostic criteria that explicitly excludes an alternative sufficient cause of the symptoms (e.g., a mental disorder or a sleep behaviour). The typical formulations identified include: “not better explained by…”, “not attributable to…”, or “not due to another…”. Content was independently reviewed and extracted by three trained coders (JAM, CG, VM) using a structured qualitative analysis protocol (Gauld, Martin, et al. 2025). Discrepancies were resolved by consensus, with arbitration from senior clinical and psychiatric experts (RL, CQ) when needed.

For each sleep disorder, the presence or absence of the NBE criterion was determined. When present, the following information was extracted: the page number in the classification manual, its position within the diagnostic criteria list (e.g., “Criterion D”), the exact wording of the formulation, the excluded condition (e.g., “a mental disorder”), and the causality wording (e.g., “not better explained”). Each NBE may include one or multiple excluded conditions. Data S1 provides the complete extraction of all NBE criteria.

### 2.4 Comparison and Visualisation of the NBE Criterion Formulation Between the ICSD‐3‐TR and the DSM‐5‐TR

The Jaccard index was computed to quantify the content overlap of NBE criteria between the ICSD‐3‐TR and the DSM‐5‐TR. The Jaccard index measures the similarity (or “overlap”) between two sets and is defined as the size of the intersection divided by the size of the union of the sets. The index ranges from 0 (no overlap) to 1 (complete overlap). Interpretation followed Evans' conventional scale: very weak (0.00–0.19), weak (0.20–0.39), moderate (0.40–0.59), strong (0.60–0.79), and very strong (0.80–1.00).

**FIGURE 1:**
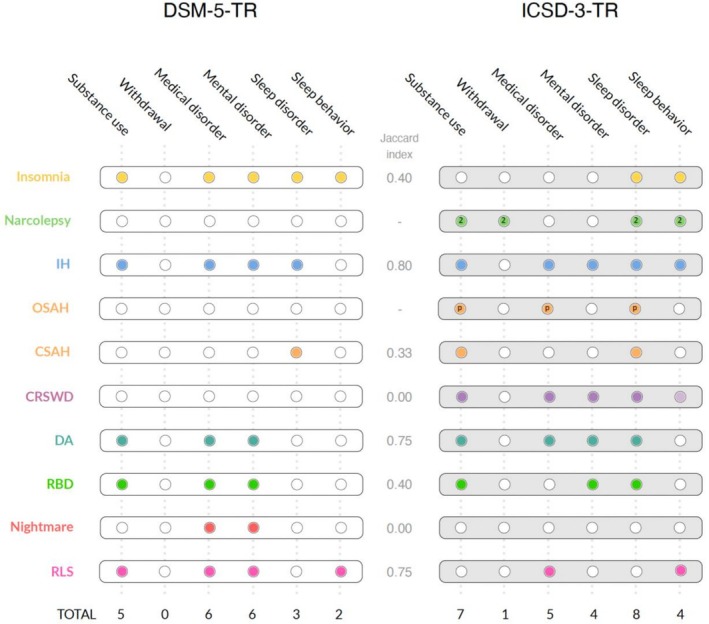
Characteristics of the target condition to be excluded in the “Not Better Explained” (NBE) criterion in the main sleep disorders retrieved in the *International Classification of Sleep Disorders, third edition revised* (ICSD‐3‐TR) and the *Sleep Disorders* section of the *Diagnostic and Statistical Manual of Mental Disorders, fifth edition revised* (DSM‐5‐TR). Each disorder is represented by a distinct colour. The presence of each characteristic is indicated by a coloured circle beneath the corresponding column. 2, Narcolepsy type 2; CRSWD, Circadian rhythm sleep–wake disorder; CSAH, central sleep apnea and hypopnea; DA, Disorders of arousal; DSM, *Diagnostic and Statistical Manual of Mental Disorders*; ICSD, *International Classification of Sleep Disorders*; IH, Idiopathic hypersomnia; OSAH, obstructive sleep apnea and hypopnea; P, paediatric OSAH; RBD, rapid eye movement sleep behaviour disorder; RLS, restless legs syndrome. For CRSWD, *sleep behaviour* is referenced solely in the ICSD‐3‐TR, specifically for shift work disorder (shown as a semi‐transparent disk).

A visualisation compared how NBE criteria are present and formulated across ten selected sleep disorders in both classifications, with emphasis on the excluded condition applied to each disorder. Figure 1. The number of each type of excluded condition across the ten sleep disorders in both classifications was calculated. For each sleep disorder, the Jaccard index was computed to assess the degree of overlap in excluded conditions between the ICSD‐3‐TR and DSM‐5‐TR.

To compare the causality formulations used in the NBE criteria, each exact wording used to indicate causality in relation to other excluded conditions was identified and counted. The consistency and frequency of use across diagnosis manuals were then compared between the two classifications (Data S1).

## 3 Results

In the ICSD‐3‐TR and DSM‐5‐TR sleep disorder classifications, the ICSD‐3‐TR includes the NBE criterion in a greater number of sleep disorders (*n* = 9 out of 10) compared to the DSM‐5‐TR (*n* = 7 out of 10). The NBE criterion was not included for OSAH and Narcolepsy Type 1 in both classifications. The overlap between the ICSD‐3‐TR and the DSM‐5‐TR is strong (Jaccard index = 0.75), suggesting convergence between these 2 classifications in the use of exclusionary logic within diagnostic criteria for sleep disorders. The only differences identified between these two classifications are as follows: the absence of the NBE criterion for CRSWDs in the DSM‐5‐TR, the absence of the NBE criterion for the Nightmare Disorder in the ICSD‐3‐TR, and the presence of the NBE criterion for Narcolepsy Type 2 and for the paediatric diagnostic criteria of OSAH in the ICSD‐3‐TR. The differences between the two classifications lie primarily in the formulation of the NBE criteria (particularly in terms of the excluded conditions and the causal formulation), rather than in their only presence or absence. Table 1.

**TABLE 1:** Summary of the presence, associated excluded condition, causality formulations of the “Not Better Explained” (NBE) criterion in the main sleep disorders retrieved in the *International Classification of Sleep Disorders, third edition revised* (ICSD‐3‐TR) and the *Sleep Disorders* section of the *Diagnostic and Statistical Manual of Mental Disorders, fifth edition revised* (DSM‐5‐TR).

Sleep disorders	DSM‐5‐TR criteria containing a NBE	ICSD‐3‐TR criteria containing a NBE	NBE in the DSM‐5‐TR	NBE in the ICSD‐3‐TR	DSM‐5‐TR formulation of the NBE criterion	ICSD‐3‐TR formulation of the NBE criterion
Chronic insomnia disorder	E. F. G. H.	C, F	✓	✓	The *sleep difficulty* **occurs despite** adequate opportunity for sleep. The *insomnia* is **not better explained by** and **does not occur exclusively during** the course of another sleep–wake disorder (e.g., narcolepsy, a breathing‐related sleep disorder, a circadian rhythm sleep–wake disorder, a parasomnia). The *insomnia* is **not attributable to** the physiological effects of a substance (e.g., a drug of abuse, a medication). Coexisting mental disorders and medical conditions **do not adequately explain** the *predominant complaint of insomnia*.	The *reported sleep/wake complaints* **cannot be explained purely** by inadequate opportunity (i.e., enough time is allotted for sleep) or inadequate circumstances (i.e., the environment is safe, dark, quiet, and comfortable) for sleep. The *sleep/wake difficulty* is **not better explained by** another sleep disorder.
Narcolepsy (NT)	—	E	—	Only NT2	—	The *hypersomnolence* and/or *MSLT* findings are **not better explained by** other causes such as insufficient sleep, obstructive sleep apnea, delayed sleep phase disorder, or the effect of medication or substances or their withdrawal.
Idiopathic hypersomnia^a^	D. E. F.	E. F.	✓	✓	The *hypersomnolence* is n**ot better explained by** and **does not occur exclusively** during the course of another sleep disorder (e.g., narcolepsy, breathing‐related sleep disorder, circadian rhythm sleep–wake disorder, or a parasomnia). The *hypersomnolence* is **not attributable to** the physiological effects of a substance (e.g., a drug of abuse, a medication). Coexisting mental and medical disorders **do not adequately explain** the predominant *complaint of hypersomnolence*.	Insufficient sleep syndrome is r**uled out** (if deemed necessary, by lack of improvement of sleepiness after an adequate trial of increased nocturnal time in bed, preferably confirmed by at least a week of wrist actigraphy). The *hypersomnolence* and/or *MSLT* findings are **not better explained** by another sleep disorder, other medical or psychiatric disorder, or use of drugs or medications.
Obstructive sleep apnea and hypopnea (OSAH)	—	C.	—	Only paediatric criteria	—	The *symptoms* are **not better explained** by another current sleep disorder, medical disorder, medication or substance use.
Central sleep apnea and hypopnea (CSAH)	B.	D.	✓	✓	The *disorder* is **not better explained** by another current sleep disorder.	The *disorder* is n**ot better explained** by another current sleep disorder, medication use (e.g., opioids), or substance use disorder.
Circadian rhythm sleep–wake disorder—General criteria	—	E.	—	✓	—	The *sleep disturbance* is **not better explained** by another current sleep disorder, medical or neurological disorder, mental disorder, medication use, or substance use disorder. “Poor sleep hygiene” is added for Shift work disorder.
Disorder of arousal—General criteria	E. F.	E.	✓	✓	The *disturbance* is **not attributable to** the physiological effects of a substance (e.g., a drug of abuse, a medication). Coexisting mental and medical disorders **do not explain** the *episodes of sleepwalking or sleep terrors*.	The *disturbance* is **not better explained** by another sleep disorder, mental disorder, medical condition, medication, or substance use.
Rapid eye movement sleep behaviour disorder (RBD)	F. G.	D	✓	✓	The *disturbance* is **not attributable to** the physiological effects of a substance (e.g., a drug of abuse, a medication) or another medical condition. Coexisting mental and medical disorders **do not explain** the *episodes*.	The *disturbance* is **not better explained** by another sleep disorder, mental disorder, medication, or substance use.
Nightmare disorder	E.	—	✓	—	Coexisting mental and medical disorders **do not adequately explain** the *predominant complaint of dysphoric dreams*.	—
Restless leg syndrome (RLS)	D. E.	B.	✓	✓	The *symptoms* in Criterion A **are not attributable** to another mental disorder or medical condition (e.g., arthritis, leg edema, peripheral ischemia, leg cramps) and are not better explained by a behavioural condition (e.g., positional discomfort, habitual foot tapping). The *symptoms* **are not attributable to** the physiological effects of a drug of abuse or medication (e.g., akathisia).	The *above features* **are not solely accounted for** as symptoms of another medical or a behavioural condition (e.g., leg cramps, positional discomfort, myalgia, venous stasis, leg edema, arthritis, habitual foot tapping).

*Note:* Legend of formatting: Sleep disorders or sleep‐related symptoms are displayed in italics; causality formulations (e.g., “not better explained”) are presented in bold; and the excluded condition is underlined. Green: present, red: not present, orange: present with condition.

^a^ Hypersomnolence disorder for the DSM‐5‐TR. Sleep disorder labels are those of the ICSD‐3‐TR.

The first difference relates to the list of excluded conditions in the NBE criteria. Figure 1. Six excluded conditions were found in the NBE criteria: “Substance use”, “Withdrawal”, “Medical disorder”, “Mental disorder”, “Sleep disorder”, and “Sleep behaviour”. The most frequently excluded conditions were “Medical disorder” and “Mental disorder” (*n* = 6 each) in the DSM‐5‐TR, and “Sleep disorder” (*n* = 8) and “Substance use” (*n* = 7) in the ICSD‐3‐TR. “Sleep behaviour” was mentioned twice as often in the ICSD‐3‐TR (*n* = 4) than in the DSM‐5‐TR (*n* = 2). “Withdrawal” was found only once in the ICSD‐3‐TR and not in the DSM‐5‐TR. Data S1. The detailed, quantified and visualised differences between the ICSD‐3‐TR and DSM‐5‐TR for each of the ten sleep disorders analysed are presented in Figure 1. The sleep disorders showing the greatest convergence in terms of excluded conditions were Idiopathic Hypersomnia, Disorders of Arousal, and RLS, whereas the lowest convergence was observed for CRSWD and CSAH. Insomnia disorder and RBD showed moderate convergence.

The second difference between the two classifications concerns the causality formulations used in the NBE criteria. Within both classifications, the following expressions were identified: “Not better explained”, “Not adequately explained”, “Cannot be explained purely”, “Not explained”, “Not attributable”, “Are not solely accounted”, “Is ruled out”, “Occurs despite”, and “Does not occur exclusively”. The ICSD‐3‐TR showed more consistent wording, with the majority of NBE criteria using “Not better explained” (*n* = 8), whereas this expression appears only 4 times in the DSM‐5‐TR. In contrast, the DSM‐5‐TR most frequently used the wording “Not attributable” (*n* = 6), but also frequently used “Not better explained” (*n* = 4) and “Not adequately explained” (*n* = 3). The causality formulations are less diverse in the ICSD‐3‐TR than in the DSM‐5‐TR. Data S1.

## 4 Discussion

As sleep medicine continues to evolve at the intersection of multiple disciplines, a more precise and transparent use of diagnostic criteria (especially those like “not better explained”—NBE criterion that require expert judgement) will be essential for aligning clinical effectiveness and epistemological precision (Gauld, Wakefield, and Micoulaud‐Franchi 2025). Thus, this first study examined systematically the diagnostic architecture of the NBE criterion across the ICSD‐3‐TR (American Academy of Sleep Medicin 2023) and of the DSM‐5‐TR (American Psychiatric Association 2022) classifications of sleep disorders. Our analysis reveals important differences between the two classifications. The ICSD‐3‐TR includes more frequent uses and more standardised terminology of the NBE criterion than the DSM‐5‐TR, which tends to use more diverse formulations of causality for exclusion. The ICSD‐3‐TR presents a broader range of excluded conditions for most of the NBE criteria (Figure 1). These excluded conditions contain mental and medical disorders, substance use, as well as other sleep disorders and sleep behaviours, indicating an integration of sleep comorbidities along with environmental and behavioural factors that may impact sleep. These differences raise important questions about how clinical reasoning is operationalised within each classification system.

### 4.1 Insomnia Disorder

The DSM‐5‐TR adopts a broad and explicit exclusion logic, stipulating that insomnia symptoms should not be better explained by a wide range of excluded conditions (including medical and mental disorders, other sleep disorders, and behavioural/environmental factors) with the sole exception of withdrawal states. In contrast, the ICSD‐3‐TR limits its exclusion criteria to other sleep disorders and behavioural/environmental factors, without explicitly invoking medical or mental disorders as alternatives. This narrower exclusion spectrum is striking because, for most other sleep disorders in the ICSD‐3‐TR, the NBE logic is generally broader than in the DSM‐5‐TR.

The rationale appears to lie in the ICSD's conceptualisation of insomnia as a disorder that often coexists with (i.e., a comorbidity logic), rather than being secondary to, medical or mental disorders (Buysse et al. 2006). In primary care settings, where insomnia is frequently managed in the context of multimorbidity and limited diagnostic resources, the absence of explicit NBE criteria regarding mental disorders in the ICSD‐3‐TR may facilitate the recognition of insomnia as a treatable condition in its own right, thereby supporting earlier intervention (e.g., cognitive‐behavioural therapy for insomnia) rather than deferral to psychiatric explanations alone. However, this approach creates a practical tension: the absence of mental disorders in the exclusion logic may reduce diagnostic precision, particularly given the strong and well‐documented comorbidity between chronic insomnia and mental disorders such as depression and anxiety disorders (Geoffroy et al. 2018; Khurshid 2018).

In specialised sleep medicine and psychiatric settings, by contrast, the DSM‐5‐TR framework provides a more explicit structure for differential diagnosis, encouraging clinicians to engage in a careful etiological appraisal of insomnia symptoms in relation to comorbid mental disorders. While this may enhance diagnostic precision, it also demands a higher level of inferential judgement and may contribute to variability in diagnostic practices across settings. When insomnia symptoms occur in the context of a mental disorder, the decision to classify them as a distinct insomnia disorder or as epiphenomena of the comorbid disorder requires indeed careful inferential reasoning (Nyhuis and Fernandez‐Mendoza 2023). NBE criteria are designed precisely to structure this reasoning, helping clinicians make justifiable diagnostic attributions (Slade and Andrews 2002). Their omission in the ICSD‐3‐TR may therefore contribute to ambiguity in research and specialised clinical practice.

This concern echoes the long‐standing debate highlighted by Edinger et al. (Edinger et al. 2004, 2011) and Buysse et al. (Buysse et al. 2006) regarding the lack of consistent operational rules for distinguishing insomnia phenotypes (Edinger et al. 1996), especially in relation to comorbidity specification (Khurshid 2018), a distinction that remains crucial for guiding intervention strategies (Geoffroy et al. 2026; Riemann et al. 2023).

Future revisions of the classifications should clarify whether insomnia is conceptualised as an autonomous disorder in the presence of comorbid disorders or whether explicit exclusion criteria are needed, potentially in combination with specifiers (as used in the DSM‐5‐TR) to balance diagnostic precision with clinical complexity.

### 4.2 Central Disorders of Hypersomnolence

Both the ICSD‐3‐TR and DSM‐5‐TR omit an NBE criterion in the diagnosis of Narcolepsy type 1 (NT1, termed simply “Narcolepsy” in the DSM‐5‐TR) (Scammell 2015). This omission is expected given the presence of a pathognomonic biomarker (hypocretin deficiency) which anchors diagnostic certainty (Dauvilliers et al. 2003; Ruoff and Rye 2016) and may further reinforce the need for accurate diagnostic classification in light of emerging orexin‐agonist therapies for NT1.

Narcolepsy type 2 (NT2) and idiopathic hypersomnia (IH in the ICSD‐3‐TR, hypersomnolence disorder—HD—in the DSM‐5‐TR) present a NBE criterion (Dauvilliers et al. 2022), but IH/HD explicitly exclude medical and mental disorders, whereas NT2 does not. This discrepancy complicates diagnostic precision for NT2 (Lammers et al. 2020), particularly when comorbid conditions create overlapping symptom presentations or influence the results of the Multiple Sleep Latency Test (MSLT) (Lopez et al. 2017). Lastly, contrary to the ICSD‐3‐TR, behavioural/environmental factors, such as insufficient sleep opportunity or irregular circadian habits, are absent in the NBE criterion of the hypersomnolence disorder of the DSM‐5‐TR. Thus, the DSM's framework may insufficiently account for modifiable external factors influencing daytime sleepiness (Biscarini et al. 2025; Vignatelli et al. 2002).

Future revisions of the classifications should aim for an approach that preserves the validity conferred by objective biomarkers while explicitly accounting for other explanatory factors in order to ensure diagnostic specificity and enable therapeutics targeting well‐defined mechanisms (Lammers et al. 2020; Gauld et al. 2020).

### 4.3 Sleep Apnea

Both the ICSD‐3‐TR and DSM‐5‐TR are notable for the absence of an NBE criterion in the diagnosis of OSAH. This omission likely reflects the central role of objective physiological markers, primarily the Apnea‐Hypopnea Index (AHI), as a diagnostic anchor. However, while AHI supports the identification of respiratory events during sleep, it does not reliably account for the presence, severity, or causal attribution of daytime symptoms (e.g., excessive sleepiness) and sleep apnea‐related disturbances (Pevernagie et al. 2020; Randerath et al. 2018; Malhotra et al. 2021; Levy et al. 2015; Veasey and Rosen 2019; Jordan et al. 2014). The correlation between AHI, arousals, and excessive daytime sleepiness is well known to be modest, particularly in mild to moderate OSAH or when apneas/hypopneas coexist with other contributing conditions (McNicholas and Pevernagie 2022; Gauld et al. 2024; Kapur et al. 2017). Moreover, AHI incompletely captures key pathophysiological dimensions of OSAH, such as hypoxic burden and sleep microstructure. Lastly, AHI values can be influenced by the method of recording and by modifiable factors such as substance use (Rosenberg and Van Hout 2014), for which an explicit exclusion would be also clinically relevant (Stanczyk et al. 2025). Interestingly, the ICSD framework has evolved over time: whereas the ICSD‐2 included an NBE criterion for adult OSAH (American Academy of Sleep Medicin 2005), this requirement was removed in the original ICSD‐3 (American Academy of Sleep Medicin 2014). However, the ICSD‐3‐TR reintroduced an exclusion criterion (American Academy of Sleep Medicin 2023), though only for paediatric OSAH, reflecting the need to clearly assess whether elevated AHI and associated symptoms in children are due to OSAH itself or to other developmental or sleep‐related disorders (Aubertin et al. 2023).

By contrast, for CSAH, both classifications adopt explicit exclusion logic, though its scope and clinical relevance differ from that observed in obstructive forms. The DSM‐5‐TR specifies that CSAH must not be better explained by another sleep disorder, whereas the ICSD‐3‐TR further incorporates substance use as an exclusion factor (Randerath et al. 2017). However, in CSAH, exclusion logic primarily concerns the interpretation of polysomnographic respiratory events rather than the attribution of daytime symptoms, which are often less prominent or less consistent (e.g., limited excessive daytime sleepiness) than in OSAH.

This highlights an important conceptual distinction: NBE criteria may operate at different levels depending on the disorder, targeting either objective markers (such as central respiratory event patterns) (Pevernagie et al. 2020; Eckert 2018) or symptom causality (Pevernagie 2021; Kapur et al. 2017). Clarifying this distinction in future revisions of the classifications could improve diagnostic precision by better aligning exclusion logic with the underlying pathophysiology and clinical expression of sleep apnea, thereby reducing inappropriate respiratory event or symptom attribution (Randerath et al. 2018).

### 4.4 Circadian Rhythm Sleep–Wake Disorders

The ICSD‐3‐TR stands out from the DSM‐5‐TR by adopting a notably comprehensive exclusion logic, explicitly stating that circadian disruptions should not be better explained by medical and mental disorders or substance use. However, despite their crucial role in CRSWDs, behavioural and environmental influences are not explicitly included as exclusion targets, except in the case of shift work disorder.

This omission is striking, as CRSWDs often result from an interplay of endogenous and exogenous factors (Coelho, Micoulaud‐Franchi, et al. 2025; Meyer et al. 2022). Circadian disruptions in CRSWDs arise from two distinct mechanisms: circadian misalignment, a mismatch between internal rhythms and external demands (often socially driven), and circadian alteration, an intrinsic failure of the physiological clock (Coelho, Martin, et al. 2025; Vetter 2020; Sack et al. 2007a, 2007b). Distinguishing these mechanisms is crucial for accurate diagnosis and targeted management (Micoulaud Franchi et al. 2025). By contrast, the DSM‐5‐TR does not include any NBE criterion clause, omitting formal consideration of alternative explanations. This absence may foster an overinclusive diagnostic process, potentially pathologising transient or situational misalignments, whereas the ICSD‐3‐TR's explicit medical and mental disorders exclusions, though incomplete, offer a more structured starting point for assessing causal plausibility.

Future revisions of the classifications should aim to refine the NBE criterion by explicitly incorporating behavioural and environmental determinants (Coelho, Martin, et al. 2025), in order to better capture true dysfunction and prevent both overmedicalisation and diagnostic ambiguity.

### 4.5 Parasomnia Disorders

Both classifications adopt exclusion criteria, but their strategies diverge in scope and emphasis. For non‐REM sleep arousal disorders (i.e., disorders of arousal—DA) (Lopez and Dauvilliers 2024; Idir et al. 2022), the DSM‐5‐TR and ICSD‐3‐TR both include medical and mental disorders as well as substance use as excluded conditions. None of the classification include electrophysiological biomarkers (Lopez et al. 2021, 2018). The ICSD‐3‐TR, however, goes further by explicitly excluding other sleep disorders, reinforcing its orientation toward etiological differentiation within the sleep medicine domain.

A similar contrast appears in the diagnosis of RBD (Neikrug and Ancoli‐Israel 2012; Arnulf 2012; Dauvilliers et al. 2018; Arnaldi et al. 2017). While both classifications include mental disorders and substance use among excluded conditions, their priorities differ: the DSM‐5‐TR emphasises exclusion of other medical disorders, whereas the ICSD‐3‐TR prioritises intra‐sleep differential diagnosis, underlining a nosological framework rooted in the internal classification of sleep disorders. The divergence becomes more marked with nightmare disorder (Aurora et al. 2010). The DSM‐5‐TR explicitly includes medical and mental disorders in its exclusion criteria, reflecting an effort to distinguish primary nightmare disorder from secondary symptoms frequently linked to trauma or mood disorders (Akkaoui et al. 2020). In contrast, the ICSD‐3‐TR provides no formal NBE criterion in its diagnostic structure.

Taken together, parasomnia disorders illustrate a broader contrast between the two classifications: the DSM‐5‐TR situates sleep disorders within the wider field of psychiatry and general medicine, emphasising exclusions beyond sleep medicine, while the ICSD‐3‐TR adopts an intra‐disciplinary approach, focusing on differential diagnosis among sleep disorders themselves. Future revisions of the classifications should carefully consider how exclusion logic reflects the underlying principles of each classification system, while exploring hybrid strategies that integrate both inter‐ and intra‐disciplinary approaches (Lopez and Dauvilliers 2024; Cicero et al. 2021; Castelnovo et al. 2018; Loddo et al. 2019).

### 4.6 Restless Legs Syndrome

The DSM‐5‐TR includes four potential alternative explanations (medical and mental disorders, substance use, and sleep behaviour) reflecting a cautious approach aimed at enhancing diagnostic specificity by controlling for confounding factors. This acknowledges that RLS‐like symptoms frequently occur in secondary contexts such as iron deficiency, renal disease, pregnancy, antipsychotic treatment, and anxiety disorders (Trenkwalder et al. 2018, 2016; Manconi et al. 2021). In contrast, the ICSD‐3‐TR adopts a more selective exclusion strategy, formally specifying only medical disorders and sleep behaviours, without explicitly mentioning mental disorders or substance‐related effects. This approach may favour diagnostic feasibility in clinical practice but risks a broader definition of RLS and potential overdiagnosis when secondary causes are present (Allen et al. 2003; Marelli et al. 2015; Walters 1995; Trenkwalder and Paulus 2010). Thus, the divergence reflects distinct orientations: DSM‐5‐TR prioritises specificity through comprehensive exclusion logic, whereas ICSD‐3‐TR emphasises simplicity at the expense of narrower differential considerations.

Future revisions of the classifications should aim for a harmonised strategy that accounts for both primary and secondary determinants or RLS, for instance by combining NBE criteria with the use of specifiers to capture cases linked to secondary conditions without conflating them with idiopathic forms.

## 5 Conclusion

The NBE criterion primarily structures diagnostic reasoning rather than symptom description, relying on clinical judgement to weigh competing causal explanations. Its use raises two major theoretical challenges: managing diagnostic hierarchies in the context of frequent comorbidities and clarifying causal attribution in the absence of explicit operational rules. A more detailed theoretical and historical analysis of exclusion logic in diagnostic reasoning is provided in the Supporting Information Data S2. This systematic content analysis reveals differences in how the NBE criterion is used and formulated across the ICSD‐3‐TR and DSM‐5‐TR. Understanding these differences is essential not only for improving diagnostic precision and managing comorbidities, but also for informing ongoing reflections on the validity, reliability, and coherence of nosological systems in both sleep medicine and psychiatry.

## Author Contributions

All authors contributed to the study conception and design. Material preparation and data collection were performed by I.L., R.L., and V.P.M. Methodology was developed by C.G., J.‐A.M.F., I.L., R.L., and V.P.M. The first draft of the manuscript was written by C.G. Analysis was performed by C.G. Editing, supervision, and reviewing were conducted by J.‐A.M.F., C.Q., and C.M.M. J.‐A.M.F. also contributed to clinical expertise and project coordination. All authors commented on previous versions of the manuscript. All authors read and approved the final manuscript.

## Funding

The authors have nothing to report.

## Ethics Statement

Ethics approval was not required for this study as it involved only the systematic content analysis of publicly available diagnostic classification manuals (ICSD‐3‐TR and DSM‐5‐TR) and did not include human or animal participants.

## Conflicts of Interest

The authors declare no conflicts of interest.

## Supporting information


**Data S1:** Supplementary Information.

**Data S2:** Supplementary Information.

## Data Availability

All data generated or analysed during this study are included in this published article and its Supporting Information.
