# Supplementary material for: Investigation of the “Not Better Explained” Diagnosis Criteria in Sleep Disorder Classifications: A Systematic Content Analysis and Critical Review
Source: J Sleep Res. 2026 Mar 13;35(4):e70327. doi: 10.1111/jsr.70327 (PMC13357857; doi:10.1111/jsr.70327)
Supplement: Supplementary file 2 — Data S2: Supplementary Information. [file JSR-35-e70327-s002.docx]

**Exclusion logic and diagnostic reasoning:**

**historical and theoretical perspectives for sleep medicine**

The NBE criterion differs from symptomatic or clinical significance criteria in that it mainly depends on the clinician’s reasoning. The “not explained” clause reflects the exhaustiveness of the diagnostic work-up, while the “better” clause relies on empirical judgment and clinical experience when weighing causal alternatives (e.g., residual excessive daytime sleepiness - rEDS in OSAH) [1]. The NBE criterion implicitly guides good clinical practice, encouraging comprehensive evaluation before diagnosis. Thus, the comparative analysis of the NBE criterion across the ICSD-3-TR [2] and DSM-5-TR [3] invites a broader reflection on the theoretical basis of exclusion logic in diagnostic reasoning in sleep medicine [4]. These results converge with philosophical discussion of causal exclusion in the field of psychiatric diagnosis [5].

Despite the longstanding role of exclusion logic in medicine, rooted in the assumption that symptoms should be explained by a single underlying cause, the explicit use of the NBE criterion was first introduced in psychiatry by Feighner et al. (1972) to operationalize diagnostic criteria for research purposes [6]. At that time, the primary aim of operationalization was to enhance the reliability of diagnostic criteria as a whole, ensuring consistent application across clinicians and studies. Within this framework, the NBE criterion had a more specific function: securing the validity of diagnostic categories in research contexts, by creating homogeneous samples and preventing overlap between disorders. As Feighner et al. stated: “Since similar clinical features and laboratory findings may be seen in patients suffering from different disorders ... it is necessary to specify exclusion criteria so that patients with other illnesses are not included in the group to be studied” [6]. This approach was later expanded in the Research Diagnostic Criteria [7] and generalized throughout DSM-III [8], extending exclusion logic beyond research into clinical practice in the DSM-IV [9, 10]. Such logic brings two interrelated theoretical challenges, managing diagnostic hierarchies and attributing causal priority, both of which reflect the broader tension between achieving clinical effectiveness and maintaining epistemological precision, a tension that is further amplified in sleep medicine by the high prevalence of comorbidities across disorders.

The first challenge lies in the hierarchical assumption embedded in exclusion logic [3, 8, 10]. Indeed, certain disorders should “take precedence” over others, a principle later termed a “dominant disorder” by Boyd (1984) [5]: “We will use the term dominant disorder to refer to a diagnosis such as major depression that takes precedence over the lesser diagnosis such as panic disorder.” This framework presumes that higher-order disorders can subsume or mimic the symptoms of lower-order ones, justifying their exclusion [5]. While conceptually coherent, such a hierarchy underestimates the frequency and clinical relevance of co-occurring disorders [9], particularly in domains like sleep medicine, where disorders frequently emerge from intertwined behavioral, medical, and psychiatric conditions [4, 11]. For example, rEDS in OSAH may accompany major depressive disorder or irregular sleep-wake patterns, raising the issue of whether it should be considered secondary or a distinct diagnosis [1]. Historically, exclusion rules sought to resolve these dilemmas through prescriptive causal hierarchies, yet this clarity often diverged from clinical reality, raising concerns about the arbitrariness of diagnostic hierarchies [12]. Clinicians can tend to overapply exclusion criteria in routine care, particularly in cases with psychiatric comorbidity, leading to a risk of therapeutic restrictions [13]. The recognition of multimorbidity in 1976 [14], highlighting the coexistence of multiple chronic disorders without privileging one as primary, has profoundly challenged this model. Unlike comorbidity, which assumes an index disorder with secondary diagnoses orbiting around it, multimorbidity promotes a patient-centered perspective in which each disorder retains diagnostic significance [15]. In sleep medicine, this shift highlights the limitations of rigid exclusion rules and the need for nosologies that reconcile diagnostic distinctiveness with the complexity of interconnected disorders. Future research should thus explore ways to use the NBE criterion in a sufficiently flexible manner to accommodate comorbidity and even multimorbidity, while also considering the role of specifiers as implemented in the DSM but not in the ICSD. The DSM uses specifiers to increase diagnostic specificity [10]. Specifiers are not mutually exclusive and allow multiple descriptors (“Specify if”) [3, 10]. For instance, the specifiers of Insomnia Disorder include: “with non-sleep disorder mental comorbidity”; “with other medical comorbidity”, “with other sleep disorder”. For sleepwalking, the specifiers include: “with sleep-related eating” and “with sleep-related sexual behavior (sexsomnia)”. Specifiers help capture clinical heterogeneity and provide information relevant for treatment. Investigating how exclusion logic and specifier logic can complement rather than compete with each other represents an important conceptual and practical direction for nosological development.

The second challenge related to diagnostic exclusion lies in the causality evaluation embedded in this exclusion logic. Although NBE criterion is intended as a rule of prudence, encouraging clinicians to consider alternative explanations before assigning a diagnosis rather than asserting causal certainty, its vague formulations (e.g., *“not attributable to”*, *“not better explained by”*) lack operational clarity, making them difficult to apply consistently [16]. Nevertheless, the wording of the NBE criterion has been carefully defined in DSM-IV and DSM-IV-TR (though removed in DSM-5), which distinguished five types of relationships: lifetime hierarchy (“criteria have never been met for…”), cross-sectional hierarchy (“criteria are not met for…”), course-related exclusion (“does not occur exclusively during the course of…”), etiological exclusion (“not due to the direct physiological effects of a substance or a general medical condition”), and differential diagnosis (“not better accounted for by…”) [10]. Such precision has not been found in the NBE for sleep disorders. This ambiguity weakens reliability, reduces diagnostic reproducibility, and complicates research validity. In the absence of shared causal models or explicit thresholds, exclusion criteria often depend on subjective judgment, raising boundary problems and questioning the coherence of current nosological systems. These concerns are amplified in psychiatry and sleep medicine, where disorders frequently share overlapping symptom networks and multifactorial determinants. Contemporary debates increasingly question whether strict categorical exclusions remain compatible with models that conceptualize symptomatology as interconnected systems rather than discrete entities [17]. In line with this shift, network-based approaches challenge the rigidity of categorical exclusions by emphasizing symptom-level interactions, suggesting new ways to conceptualize sleep disorders and refine classification [4]. Moreover, in sleep medicine, causal attribution related to behavioral and environmental factors, such as insufficient sleep opportunity or irregular circadian habits, are clinically quantifiable [1]. However, assessing their respective causal weight for symptoms remains difficult. Causal attribution often relies on expert judgment, embodying implicit theories of causation and disorder boundaries, which introduces subjectivity and compromises both research validity and clinical consistency. Future research should prioritize the development of explicit causal models, combining data-driven approaches (e.g., network or dynamical modeling) with expert-informed frameworks [18, 19], to provide transparent and operationalizable exclusion criteria that preserve clinical interpretability while ensuring reproducibility.

In conclusion, the presence of exclusion criteria reflects a profound epistemic tension between two competing imperatives: validity and utility. On one hand, exclusion rules aim to enhance diagnostic validity by specifying clear boundaries between disorders and avoiding conceptual overlap. On the other, such exclusion rules should remain clinically useful, supporting decision-making in complex presentations rather than introducing ambiguity or rigidity. Clinicians therefore require rigorous models while retaining criteriological flexibility, allowing them to engage with uncertainty both prudently and rigorously.

**References**

1. Barateau, L., S. Baillieul, C. Andrejak, E. Bequignon, P. Boutouyrie, Y. Dauvilliers, F. Gagnadoux, P.A. Geoffroy, J.A. Micoulaud-Franchi, D. Montani, C. Monaca, M. Patout, J.L. Pepin, P. Philip, C. Pilette, R. Tamisier, W. Trzepizur, D. Jaffuel, and I. Arnulf, *Guidelines for the assessment and management of residual sleepiness in obstructive apnea-hypopnea syndrome: Endorsed by the French Sleep Research and Medicine Society (SFRMS) and the French Speaking Society of Respiratory Diseases (SPLF).* **Respir Med Res**, 2024. 86: p. 101105.

2. American Academy of Sleep Medicin, *International Classification of Sleep Disorders : ICSD-3-TR.* 2023.

3. American Psychiatric Association, *Diagnostic and Statistical Manual of Mental Disorder, 5th ed revised (DSM-5-TR)*. 2022, Washington, DC: American Psychiatric Association.

4. Gauld, C., J.C. Wakefield, and J.A. Micoulaud-Franchi, *Proposing a definition for sleep disorders: An epistemological review.* **Sleep Med Rev**, 2025. 79: p. 102028.

5. Boyd, J.H., J.D. Burke, Jr., E. Gruenberg, C.E. Holzer, 3rd, D.S. Rae, L.K. George, M. Karno, R. Stoltzman, L. McEvoy, and G. Nestadt, *Exclusion criteria of DSM-III. A study of co-occurrence of hierarchy-free syndromes.* **Arch Gen Psychiatry**, 1984. 41(10): p. 983-9.

6. Feighner, J.P., E. Robins, S.B. Guze, R.A. Woodruff, Jr., G. Winokur, and R. Munoz, *Diagnostic criteria for use in psychiatric research.* **Arch Gen Psychiatry**, 1972. 26(1): p. 57-63.

7. Spitzer, R.L., J. Endicott, and E. Robins, *Research diagnostic criteria: rationale and reliability.* **Arch Gen Psychiatry**, 1978. 35(6): p. 773-82.

8. American Psychiatric Association, *Diagnostic and statistical manual of mental disorders : DSM III.* 1980.

9. Slade, T. and G. Andrews, *Exclusion criteria in the diagnostic classifications of DSM-IV and ICD-10: revisiting the co-occurrence of psychiatric syndromes.* **Psychol Med**, 2002. 32(7): p. 1203-11.

10. American Psychiatric Association, *Diagnostic and Statistical Manual of Mental Disorder, 4th ed (DSM-IV).* 1994.

11. Pevernagie, D., *Future Treatment of Sleep Disorders: Syndromic Approach Versus Management of Treatable Traits?* **Sleep Med Clin**, 2021. 16(3): p. 465-473.

12. Zimmerman, M., J.B. McGlinchey, I. Chelminski, and D. Young, *Diagnosing major depressive disorder V: applying the DSM-IV exclusion criteria in clinical practice.* **J Nerv Ment Dis**, 2006. 194(7): p. 530-3.

13. Ronconi, J.M., B. Shiner, and B.V. Watts, *Inclusion and exclusion criteria in randomized controlled trials of psychotherapy for PTSD.* **J Psychiatr Pract**, 2014. 20(1): p. 25-37.

14. Brandlmeier, P., *[Multimorbidity among elderly patients in an urban general practice].* **ZFA (Stuttgart)**, 1976. 52(25): p. 1269-75.

15. Feinstein, A.R., *The Pre-Therapeutic Classification of Co-Morbidity in Chronic Disease.* **J Chronic Dis**, 1970. 23(7): p. 455-68.

16. Kendler, K.S., *Historical precedents for the DSM-III bereavement exclusion criteria for major depression.* **Psychol Med**, 2018. 48(16): p. 2794-2803.

17. Gauld, C., R. Lopez, C. Morin, P.A. Geoffroy, J. Maquet, P. Desvergnes, A. McGonigal, Y. Dauvilliers, P. Philip, G. Dumas, and J.A. Micoulaud-Franchi, *Symptom network analysis of the sleep disorders diagnostic criteria based on the clinical text of the ICSD-3.* **J Sleep Res**, 2022. 31(1): p. e13435.

18. Martin, V.P., R. Lopez, J.A. Micoulaud-Franchi, and C. Gauld, *Disappearance and dissemination of sleep symptoms: the importance of sleep medicine expertise for psychiatry. A comment on Forbes et al.* **Psychol Med**, 2024. 54(12): p. 1-3.

19. Gauld, C., T.F. Blanken, L. Klintwall, and J.A. Micoulaud-Franchi, *Introducing Perceived Causal Networks in Sleep Medicine.* **J Sleep Res**, 2025: p. e70035.
